# Reprocessable Vitrimers From Post‐Consumer Polyolefin Blends: Elucidating Phase‐Selective Dynamic Networks

**DOI:** 10.1002/marc.70373

**Published:** 2026-07-20

**Authors:** Subhaprad Ash, Rishi Sharma, Li Xie, Muhammad Rabnawaz

**Affiliations:** ^1^ School of Packaging Michigan State University East Lansing Michigan USA; ^2^ Department of Chemistry Michigan State University East Lansing Michigan USA

**Keywords:** polyolefin vitrimers, post‐consumer plastic waste, phase‐selective crosslinking, upcycling

## Abstract

Reprocessable and recyclable polyolefin (PO) vitrimers can be used as alternatives to conventional non‐recyclable thermosets. While recent studies have successfully demonstrated dynamic network formation in virgin POs, the application of dynamic covalent chemistry in post‐consumer mixed POs remains unexplored. Herein, we report the first reactive melt extrusion approach that utilizes nitroxide‐mediated silyl ether chemistry to produce post‐consumer high‐density polyethylene (HDPE) and polypropylene (PP) blended vitrimers. Key findings show that the fully formulated 75:25 wt./wt. HDPE/PP vitrimer creates a covalently crosslinked network with a storage modulus of >1 MPa at 220°C. Thermomechanical benchmarking against a permanent epoxy‐ester thermoset reveals that the upcycled vitrimer combines thermoset‐like dimensional stability above 180°C with dynamic melt‐reprocessability, albeit with a modulus above its melting transition that is lower than the permanent network. For the first time, 2D wideline separation (2D WISE) solid‐state NMR was used to qualitatively validate highly phase‐selective crosslinking. This is the first study that establishes vitrimer formation from mixed post‐consumer PO blends, thus providing a scalable upcycling pathway for municipal solid waste.

## Graphical abstract

Reactive melt extrusion utilizing nitroxide‐mediated silyl ether chemistry transforms post‐consumer high‐density polyethylene and polypropylene blends into robust vitrimers. Featuring highly phase‐selective crosslinking, these upcycled materials combine thermoset‐like dimensional stability at elevated temperatures with dynamic melt‐reprocessability. This strategy establishes a scalable, innovative pathway to upcycle mixed municipal polyolefin waste into sustainable thermoset alternatives.

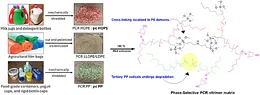

## 1 Introduction

The objective of this work is to synthesize reprocessable vitrimers from heterogeneous post‐consumer polyolefin (PO) waste blends, polyethylene (PE) and polypropylene (PP) [1, 2], that achieve the mechanical strength of thermosets while retaining the desired melt reprocessability. This objective is critical because POs constitute the vast majority of municipal solid waste globally [3]; yet, traditional mechanical recycling of these mixed streams is impeded by the severe thermodynamic immiscibility, leading to inevitable thermal degradation, poor mechanical retention, and the continuous downcycling of valuable plastic resources [4, 5, 6].

POs constitute more than half of all generated plastic waste, yet their recovery is severely limited by conventional mechanical recycling [7, 8, 9]. Because PE and PP are thermodynamically immiscible, blending these post‐consumer streams typically results a high degree of phase separation and significantly degraded mechanical properties [10, 11]. To resolve the trade‐off between processability and structural performance, dynamic chemical crosslinking has emerged as a highly effective strategy for transforming these thermoplastics into covalent adaptable networks [7, 12, 13]. Previously, pioneering researchers such as Leibler et al. [14] introduced the concept of vitrimers, and more recent studies have successfully utilized co‐polymerization [15] and dynamic covalent chemistries such as transesterification [16, 17, 18, 19, 20, 21, 22, 23], boronic ester exchange [24, 25, 26, 27], disulfide exchange [28, 29, 30, 31, 32, 33], urethane exchange [34, 35, 36, 37], and silyl ether exchange [8, 38, 39, 40, 41, 42] to achieve reprocessable crosslinked networks in polymer systems. However, these previous approaches have been limited by their almost exclusive reliance on pristine, homogeneous model materials. When applied to real post‐consumer waste [2], these methods are severely hindered by phase incompatibility [43, 44], contamination, and competing radical degradation pathways. A notable example of such a pathway is the rapid macroscopic β‐scission that occurs within polypropylene domains during high‐temperature extrusion [45], which ultimately prevents the formation of a continuous, stress‐bearing network. Specifically, while PE domains readily undergo crosslinking, PP radicals formed during melt extrusion are highly susceptible to chain rearrangement and global chain scission, resulting in unsaturated fragments and a drastic reduction in molecular weight [8, 45]. Consequently, a significant research gap remains in designing robust vitrimer systems that are capable of accommodating the inherent heterogeneity of mixed municipal solid waste despite the existence of a few studies on post‐consumer resins [7]. Although some methods exist for pure polymers [10, 45], there is currently a lack of facile, closed‐loop upcycling strategies that would be capable of overcoming the degradative β‐scission in PP‐rich blends to produce high‐performance, mixed PO vitrimers without requiring prior sorting or separation.

We began this work with a simple question: can mixed post‐consumer polyethylene and polypropylene be converted into vitrimers without first separating them, even with all the additives, contaminants, and pigments that come with the post‐consumer resin (PCR)? To find out, we performed reactive melt extrusion using nitroxide‐mediated silyl ether chemistry in PE/PP PCR. We demonstrated that the nitroxide radical scavenger (2,2,6,6‐tetramethyl‐4‐piperidinol) suppresses the side reactions and facilitates dynamic silyl ether network formation, thus providing a highly practical and sustainable pathway for upcycling mixed plastic waste to a vitrimer.

## 2 Experimental Section

### 2.1 Materials and Methods

To accurately simulate the heterogeneous nature of municipal solid waste (MSW), authentic post‐consumer polyolefin (PO) resins were sourced and processed prior to reactive extrusion. High‐density polyethylene (HDPE) was obtained from discarded milk jugs and detergent bottles and mechanically shredded into flakes. Linear low‐density and low‐density polyethylene (LLDPE/LDPE) were sourced from agricultural film bags, which were subsequently cut and pelletized via extrusion to ensure consistent feeding. Polypropylene (PP) was derived from a mixture of food‐grade containers, yogurt cups, and rigid bottle caps, and was similarly mechanically shredded into flakes. These PCRs were then blended to formulate the targeted blends to be used for the vitrimer synthesis. All chemical reagents were used as received without prior purification. Vinyltrimethoxysilane (VTMS, 98%), dicumyl peroxide (DCP, 98%), and 2,2,6,6‐tetramethyl‐4‐piperidinol (T, 98%) were procured from Sigma–Aldrich (Milwaukee, WI), while bis[3‐(trimethoxysilyl)propyl]amine (BTMSPA, 97%) was acquired from TCI America.

### 2.2 Reactive Melt Extrusion of Vitrimers

The compositional formulations for the synthesized vitrimers are detailed in Table 1. The polymer matrices and chemical reagents were physically pre‐mixed prior to being fed into a 15 cc DSM micro‐extruder equipped with co‐rotating conical twin screws. Reactive melt blending was conducted under a nitrogen atmosphere at a barrel temperature of 190°C with a screw speed of 100 rpm. A residence time of 4 min was utilized to ensure that the torque of the system reached a steady‐state equilibrium. Immediately following extrusion, the molten extrudates were transferred to an Xplore IM5.5 (#0802) injection molding machine to fabricate test specimens for dynamic mechanical, tensile, and impact analyses. The injection molder was operated at a melt temperature of 190°C, an injection pressure of 5 bar, and a mold temperature of 35°C.

**TABLE 1:** Formulations (mol %) of the synthesized vitrimers.

Sample	HDPE (mol %)	PP (mol %)	Dicumyl peroxide DCP (mol %)	Vinyltrimethoxysilane V (mol %)	Bis[3‐(trimethoxysilyl)propyl]amine BTMSPA (mol %)	2,2,6,6‐Tetramethl‐4‐piperidinol T (mol %)
PCR HDPE Series						
pc HDPE	100.00	0.00	0.00	0.00	0.00	0.00
pc HDPE+V	98.99	0.00	0.02	0.49	0.50	0.00
pc HDPE+V+T	98.96	0.00	0.02	0.50	0.50	0.03

PCR HDPE rich blend						
pc H_75_‐P_25_	81.82	18.18	0.00	0.00	0.00	0.00
pc H_75_‐P_25_+V	74.69	16.60	0.02	0.49	0.50	0.00
pc H_75_‐P_25_+V+T	74.67	16.59	0.02	0.50	0.50	0.03

PCR PP rich blend						
pc H_25_‐P_75_	18.18	81.82	0.00	0.00	0.00	0.00
pc H_25_‐P_75_+V	16.60	74.69	0.02	0.49	0.50	0.00
pc H_25_‐P_75_+V+T	16.47	74.69	0.02	0.50	0.50	0.03

PCR PP Series						
pc PP	0.00	100.00	0.00	0.00	0.00	0.00
pc PP+V	0.00	98.99	0.02	0.49	0.50	0.00
pc PP+V+T	0.00	98.96	0.02	0.50	0.50	0.03

For nomenclature, **pc H_75_‐P_25_+T** indicates a post‐consumer blend containing 75 wt.% HDPE and 25 wt.% PP. The reactive system includes 0.5 mol% vinyltrimethoxysilane (**V**) as the grafting agent, 2,2,6,6‐tetramethyl‐4‐piperidinol (**T**) as the nitroxide radical scavenger, dicumyl peroxide (**DCP**) as the radical initiator, and bis[3‐(trimethoxysilyl)propyl]amine (**BTMSPA**) as the secondary amine crosslinker.

During the reactive extrusion process at 190°C, the condensation reaction between the methoxysilane groups inherently generates methanol as a byproduct. Because this processing temperature significantly exceeds the boiling point of methanol (64.7°C), the generated alcohol immediately volatilized and was safely vented from the extruder during the residence time.

### 2.3 Fourier‐Transform Infrared Spectroscopy (FTIR)

Chemical structural modifications were monitored utilizing attenuated total reflectance Fourier‐transform infrared (ATR‐FTIR) spectroscopy. Spectra were acquired on a Jasco FTIR‐6600 spectrometer equipped with an ATR Pro‐One module utilizing a 28° Michelson interferometer. Data collection spanned a wavenumber range of 400–4000 cm^−1^, with the number of scans determined by the instrument's auto‐scan mode. All resulting spectral data were analyzed using Spectra Manager software developed by JASCO Inc.

### 2.4 Dynamic Mechanical Analysis (DMA)

Thermomechanical behavior was evaluated utilizing an RSA‐G2 DMA analyzer (TA Instruments). Injection‐molded rectangular specimens (12.5 mm × 6.25 mm × 3.25 mm) were tested in the tensile mode at a constant frequency of 1 Hz and an applied strain of 0.01%. Following a 5‐min isothermal equilibration at 40°C, temperature sweeps were conducted from 40°C to 220°C at a heating rate of 2°C min^−1^. Due to the onset of macroscopic melt flow, uncrosslinked PO controls (e.g., pure HDPE) were only analyzed up to 140°C.

### 2.5 Solid‐State Nuclear Magnetic Resonance (NMR) Spectroscopy

All NMR data were acquired on a Bruker 400 MHz Avance Neo spectrometer equipped with a 3.2 mm HCN probe at 298 K. The rotor spinning speed was maintained at 10 kHz for 2D ^1^H−^13^C wideline separation (WISE) NMR and 20 kHz for 1D cross‐polarization magic‐angle spinning (CP‐MAS).

2D WISE NMR experiments were performed using a heteronuclear correlation (HETCOR)‐style pulse sequence [46]. The pulse program utilized was hXHETCOR.cp with ZGOPTNS = ‐Dnodec for WISE‐style HETCOR [47]. The cross‐polarization contact time was set to 100 µs to minimize spin diffusion and accurately capture the mobility of the distinct phases. Generally, the WISE NMR data was acquired with 128 points in the t_1_ (^1^H) dimension and 200 data points in the t_2_ (^13^C) dimension. The spectral widths were 8.2 and 160 kHz in the t_1_ and t_2_ dimensions, respectively. The recycle delay was 2 s and the number of scans was 800. A typical WISE experiment lasted about 2 days and 10 h.

1D CP MAS data was acquired with a recycle delay of 2 s and a CP contact time of 2 ms. The number of scans was 6000.

### 2.6 Differential Scanning Calorimetry (DSC)

Thermal transitions were characterized using a TA Instruments DSC 2500. During each measurement, approximately 8.0 ± 1.0 mg of sample was sealed in a standard aluminum pan with a lid. Measurements were conducted under a steady nitrogen purge (100 mL min^−1^). To erase prior thermal history, the samples were initially heated from 25°C to 200°C at a ramp rate of 10°C min^−1^, followed by cooling at the same rate. Thermal data were recorded during the subsequent second heating cycle from 25°C to 200°C.

### 2.7 Ultimate Tensile Testing

Mechanical tensile properties were assessed following ASTM D638 [48] guidelines using an Instron 5565 P6021 universal testing machine. Prior to testing, the injection‐molded Type V specimens (gauge dimensions: 12.5 mm length × 3.25 mm width × 3.25 mm thickness) were conditioned at 23°C for 48 h. Tests were conducted at a constant crosshead extension rate of 10 mm min^−1^. For each formulation, five replicate specimens were tested to determine the average tensile strength and standard deviation.

### 2.8 Thermogravimetric Analysis (TGA)

The thermal degradation profiles of the materials were evaluated utilizing a TA Discovery 550 Thermogravimetric Analyzer. Samples weighing 8.0 ± 1.0 mg were placed in aluminum pans and heated from 25°C to 600°C. A constant heating rate of 10°C min^−^^1^ was applied under a nitrogen atmosphere to record weight loss as a function of temperature.

## 3 Results and Discussion

### 3.1 Hypothesis and Reaction Pathway

This study was fundamentally hypothesis‐driven: if virgin PO blends can be successfully converted into covalently adaptable networks as we established in our previous studies [8], then unseparated post‐consumer resins (PCR) should exhibit comparable reactivity. Our rationale is that, although PCR contains contaminants and additives (stabilizers, etc.), similar additives are also present in commercially produced virgin pellets today. Therefore, based on the successful conversion of virgin pelletized PP/PE blends that have been achieved in our previous work, we propose that PCR materials may also be converted into vitrimers.

**FIGURE 1:**
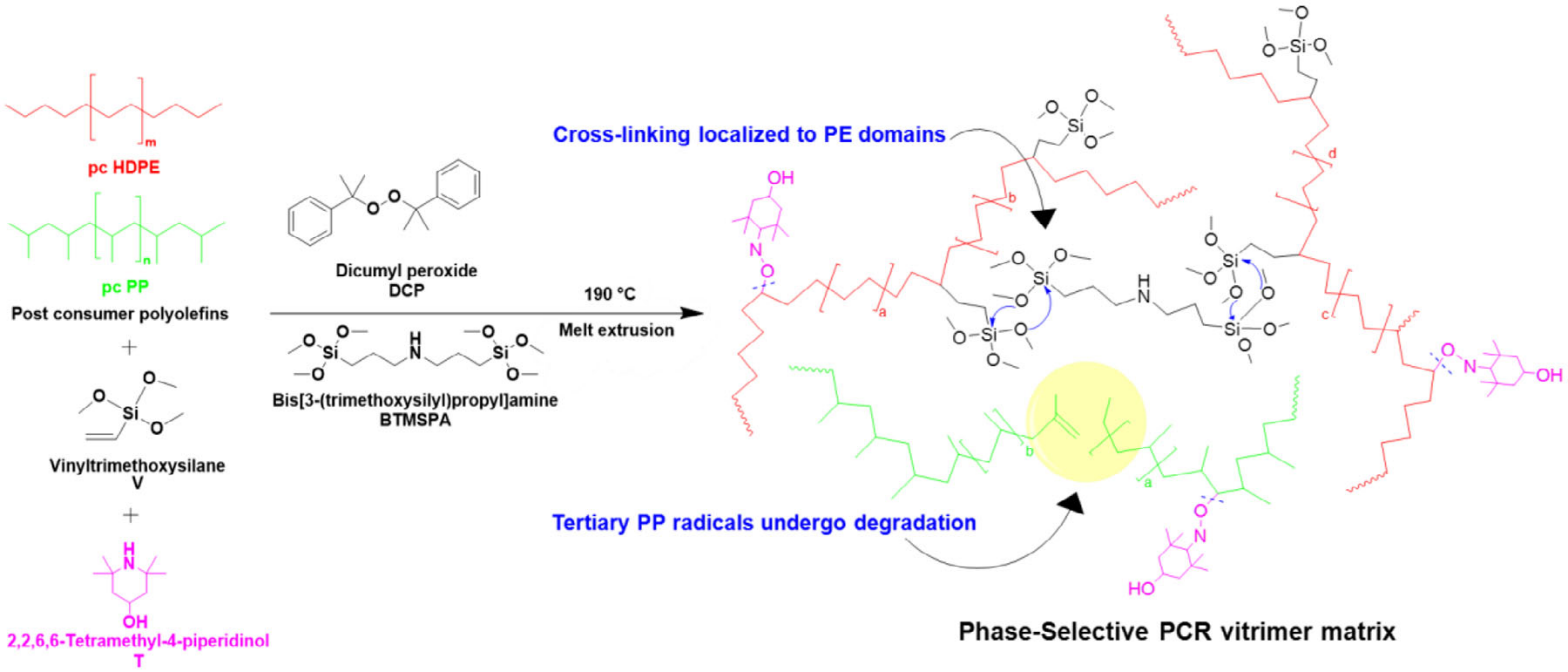
Mechanistic illustration of the radical‐mediated functionalization of mixed post‐consumer POs. The schematic highlights successful nitroxide‐mediated silyl ether crosslinking in polyethylene (PE) architectures, contrasted with the inherently dominant β‐scission and fragmentation pathways in polypropylene (PP) domains.

To verify our hypothesis, reactive melt extrusion was applied directly to the mixed PCR streams. The generalized reaction pathway for the fabrication of these dynamic networks is depicted in Figure 1. Crucially, while PE and PP belong to the exact same PO family, they exhibit fundamentally distinct chemical responses to the radical‐mediated vitrimer formation process. As illustrated in the scheme provided in Figure 1, the secondary carbons present in the PE backbone readily support the bimolecular grafting of V, paving the way for robust silyl ether crosslinking. Conversely, the sterically hindered tertiary carbons in the PP backbone heavily favor unimolecular β‐scission, promoting rapid chain fragmentation rather than continuous network formation. The introduction of the nitroxide radical scavenger (T) mediates this disparate behavior, suppressing uncontrolled dead‐end coupling and macroscopic chain scission to maximize grafting efficiency across the heterogeneous blend. This reactive extrusion methodology was applied to synthesize dynamic networks from both pure post‐consumer feedstocks (pc HDPE and pc PP) and their mixed binary blends at 75:25 and 25:75 weight ratios (designated as the pc H‐P series). A comprehensive breakdown of these specific formulations is provided in Table 1.

### 3.2 FT‐IR Confirmation of Silyl Ether Network Formation and Confirmation of Grafting Efficiency via Extraction Analysis

Fourier‐transform infrared (FT‐IR) spectroscopy was utilized to verify the chemical modifications and structural composition of the synthesized dynamic networks. The successful grafting and crosslinking of the polymer chains were evidenced by the emergence of a distinct absorption band at 1092 cm^−1^, which is characteristic of Si─O stretching vibrations [49] and confirms the incorporation of silyl ether linkages within the matrix (Figure S1).

The structural integrity of the primary PO backbones was maintained post‐extrusion, as indicated by the intense absorption peaks at 2919 and 2850 cm^−1^. These bands correspond to the fundamental asymmetric and symmetric C─H stretching modes that are prevalent in both polyethylene and polypropylene. Additionally, the typical C─H rocking deformations for these aliphatic chains were observed as a doublet at 730 and 720 cm^−1^. Furthermore, FT‐IR spectroscopy proved to be an effective tool for qualitatively tracking the compositional ratios of the distinct polymer phases within the heterogeneous blends. The absorption peak at 1376 cm^−1^, which is specifically assigned to the symmetric deformation of methyl groups in PP, served as a reliable marker. As illustrated in Figure S1, the intensity of this 1376 cm^−1^ signal scales proportionally with the increased loading of PP in the respective H‐P blend formulations. Analogous spectral signatures and proportional peak variations were also observed for the broader post‐consumer PO mixtures (provided in Figure S2).

To confirm the degree of covalent grafting of the Vinyltrimethoxysilane, FT‐IR analysis was performed on the polymer blends before and after extraction in xylene at 140°C for 2 h followed by oven‐drying at 75°C for 24 h. (Figure S3). The relative silane concentration was quantified using the absorbance ratio of the Si─O─Si or Si─O─C stretching vibration at 1092 cm^−1^ to the CH_2_ bending vibration at 1461 cm^−1^ (A_1092_/A_1461_). The 1461 cm^−1^ peak was strategically employed as an internal reference because it remains stable across the complex, heterogeneous composition of the post‐consumer PE and PP matrices, unlike the highly variable 1377 or 720 cm^−1^ peaks. The extraction data reveal higher grafting and crosslinking efficiencies within the nitroxide‐mediated systems. An average reduction of 18.5% in the relative silane ratio was observed following extraction (Table S1). Crucially, this reduction was far more pronounced in the PP‐rich formulations, directly confirming that a substantial portion of the silane is lost alongside highly fragmented, soluble polymer chains generated via unmitigated β‐scission reactions. Conversely, the fully formulated pc HDPE‐rich vitrimer system (pc H_75_‐P_25_+V+T) exhibits near‐total retention of the silane ratio post‐extraction. This chemical evidence of a highly grafted, insoluble network aligns with the macroscopic thermomechanical performance observed in the DMA results, confirming that nitroxide mediation enhances the dynamic linkages into a stable architecture.

### 3.3 Thermomechanical Properties of Vitrimers and Control Blends (Dynamic Mechanical Analysis)

**FIGURE 2:**
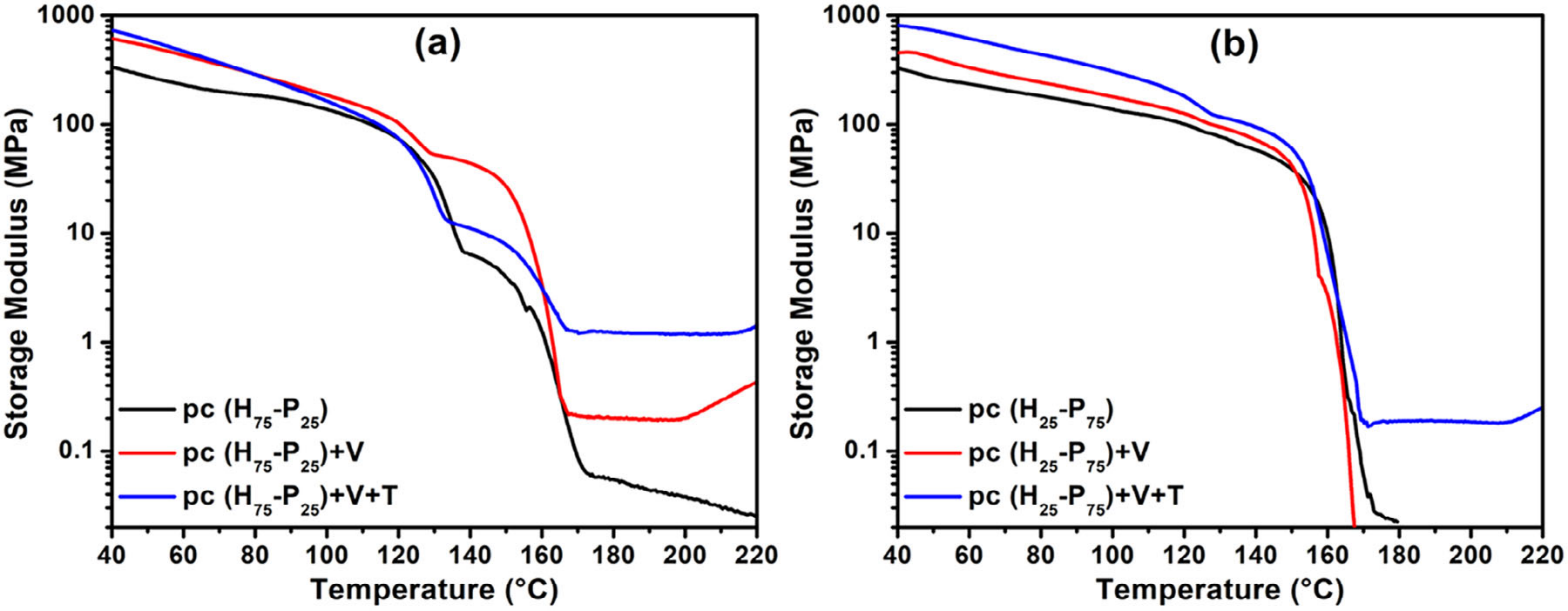
Thermomechanical analysis of the synthesized dynamic networks via dynamic mechanical analysis (DMA). Storage modulus (***E'***) as a function of temperature is plotted for (a) 75:25 pc HDPE/PP blends, and (b) 25:75 pc HDPE/PP blends. Each panel compares the neat post‐consumer control (black) to the VTMS‐grafted system (+V, red) and the fully formulated vitrimer containing the nitroxide scavenger (+V+T, blue), highlighting the presence or absence of a high‐temperature rubbery plateau.

Dynamic mechanical analysis (DMA) was utilized to probe the thermomechanical transitions and confirm the formation of the covalently crosslinked vitrimer networks. A defining characteristic of a successful thermoset‐like material is the manifestation of a stable rubbery plateau, where the storage modulus (***E'***) remains relatively constant at temperatures well above the crystalline melting points (***T*_m_**) of the constituent polymers. The DMA profiles of the post‐consumer formulations are presented in Figure 2 and Figure S4.

Pure pc HDPE systems are shown in Figure S4a. A neat pc HDPE control should exhibit typical thermoplastic behavior, characterized by a sharp decline in the storage when the temperature is increased above its melting transition (∼135°C). The storage modulus drops to approximately 0.1 MPa. Ideally, a pure, uncrosslinked thermoplastic melt would approach a modulus of zero, but the presence of inorganic fillers or highly branched impurities that are common in post‐consumer waste likely provides a minor degree of residual melt strength. In stark contrast, both the grafted (+V) and fully formulated (+V+T) pc HDPE vitrimers establish a robust rubbery plateau, sustaining a storage modulus of approximately 1.2 MPa up to 220°C. The similarity between the +V and +V+T curves suggests that for highly reactive polyethylene, where secondary macroradicals naturally tend toward rapid recombination and crosslinking, the addition of the nitroxide radical scavenger (T) is not strictly necessary to achieve a dense network.

The storage moduli data for HDPE‐rich blends (75:25 HDPE/PP) is shown in Figure 2a. The DMA profiles for the mixed blends clearly reveal two distinct drops in the storage modulus, corresponding to the sequential melting of the thermodynamically immiscible HDPE (∼130°C) and PP (∼165°C) phases. The unmodified control blend eventually collapses to 0.05 MPa at 180°C and to 0 MPa at 220°C, confirming the absence of a chemical network. When silane, peroxide, and the BTMSPA crosslinker are present (in the +V system), the vitrimer achieves a weak plateau (∼0.2 MPa). However, the inclusion of the nitroxide (+V+T) triggers an impressive enhancement, elevating the high‐temperature storage modulus to over 1 MPa. This substantial increase in structural robustness is directly attributed to the reversible radical trapping mechanism of the nitroxide mediator. The grafting process competes heavily with rapid free‐radical degradation (primarily β‐scission within the PP domains), which prematurely terminates network growth. The introduction of the nitroxide (+T) establishes a dynamic activation/deactivation equilibrium. The nitroxide rapidly reacts with the polymer macroradicals to form a dormant, temporarily bonded species that reversibly dissociates. By keeping the instantaneous concentration of active radicals extremely low, the nitroxide effectively suppresses the fast chain‐scission pathways. Concurrently, the reversible nature of the bond keeps the macroradicals “alive” long enough to maximize the slower silane grafting efficiency. This controlled radical environment allows the pre‐existing BTMSPA crosslinker to weave a significantly denser and more homogeneous covalent network, leading to the elevated thermomechanical properties observed at the high‐temperature plateau.

PP‐rich blends (25:75 HDPE/PP) storage moduli data are shown in Figure 2b. As the concentration of PP increases, network formation becomes significantly more challenging. Both the control and the +V system exhibit a catastrophic drop in their storage modulus near 170°C, indicating flow behavior that is typical of uncrosslinked melts. Interestingly, the fully formulated (+V+T) system manages to maintain a slight, albeit weak, crosslinked architecture, as evidenced by a modest plateau near 0.2 MPa. This further confirms that nitroxide‐mediated radical control is essential to extract any degree of crosslinking in environments that are heavily populated by PP.

In the pure pc PP systems (depicted in Figure S4b) none of the formulations, regardless of the presence of V or T were capable of sustaining a rubbery plateau, with all samples demonstrating complete macroscopic flow above 170°C. This failure to form a network is rooted in the fundamental radical chemistry of PP. Under radical initiation at elevated extrusion temperatures, the tertiary carbon centers in the PP backbone form highly sterically hindered and relatively stable macroradicals. Rather than participating in the desired bimolecular grafting reaction with VTMS, these tertiary radicals undergo rapid, unimolecular β‐scission. This chain fragmentation pathway degrades the polymer into lower molecular weight, unsaturated species, thereby completely impeding the development of a continuous silyl ether network.

### 3.4 Molecular‐Level Validation of Phase‐Selective Crosslinking (Solid‐State NMR)

While dynamic mechanical and thermal analyses provided macroscopic evidence of phase‐selective network formation, solid‐state nuclear magnetic resonance (NMR) spectroscopy was employed to obtain definitive, molecular‐level corroboration. To confirm that the silyl ether network localizes exclusively within the polyethylene domains, 2D wideline separation (2D WISE) NMR was performed on the mixed polyolefin system simulating municipal solid waste (control PO blend) and its corresponding vitrimer (PO+V+T vitrimer). PO blend comprises of 27.7% HDPE, 36.3% LLDPE/LDPE, and 36.0% PP by weight.

The 1D ^13^C Cross‐Polarization Magic Angle Spinning (CP‐MAS) spectra of the control blend and the formulated vitrimer (Figure S5) provide the foundational structural resolution required to interpret the 2D data. Distinct chemical shifts were assigned to the discrete polymer phases: the PP domains are identified by resonances at 44.5 ppm (methine CH), 26.1 ppm (methylene CH_2_), and 21.2 ppm (methyl CH_3_). The PE domains are distinguished by a dominant peak at 32.6 ppm (crystalline methylene CH_2_) with a prominent shoulder at ∼30.7 ppm corresponding to the amorphous PE fraction.

**FIGURE 3:**
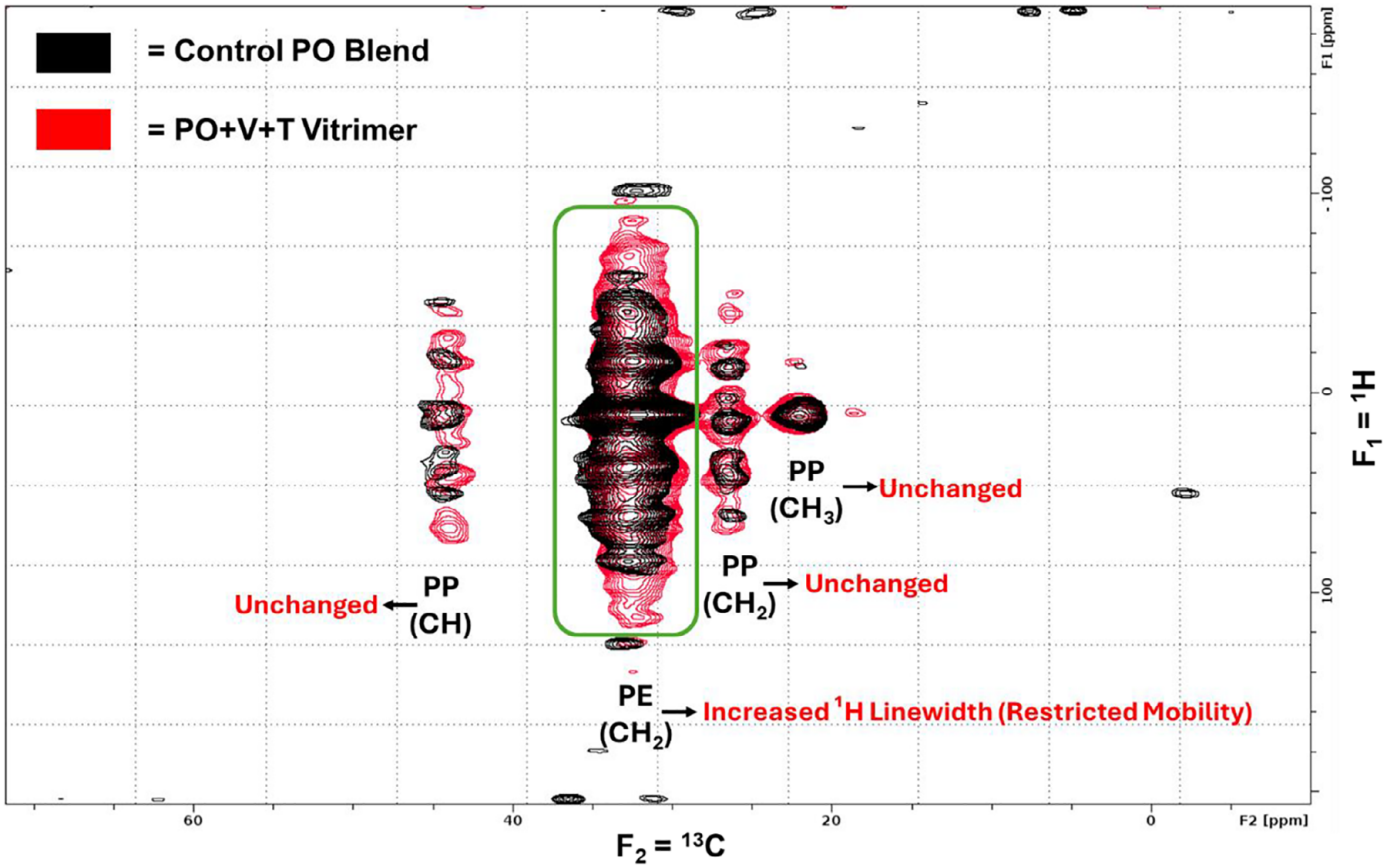
2D wideline separation (WISE) NMR spectra correlating structural phases (*F_2_*, ^13^C dimension) with chain mobility (*F_1_*, ^1^H dimension). The overlay compares the uncrosslinked control PO blend (black contours) and the upcycled PO+V+T vitrimer (red contours). The drastic increase in ^1^H linewidth exclusively at the PE (CH_2_) resonance indicates restricted segmental mobility.

To correlate these distinct structural phases with their dynamic chain mobility, 2D WISE NMR spectroscopy was utilized. In this technique, the *F_2_* axis represents the ^13^C chemical shift (structural identity), while the *F_1_* axis displays the ^1^H wideline profile (segmental mobility). Because strong ^1^H–^1^H dipolar couplings in rigid, restricted segments result in broad proton lines, whereas highly mobile segments experience rapid motional averaging (yielding narrow lines) [50], the *F_1_* linewidth serves as a direct probe of the crosslinking density. An overlay of the 2D WISE spectra for the uncrosslinked control PO (black contours) and the fully formulated silyl ether PO vitrimer (red contours) unequivocally reveals the phase‐selective nature of the reactive extrusion process (Figure 3). The most profound dynamic shift occurs within the PE region at 30–33 ppm. In the *F_1_* dimension, the vitrimer sample exhibits a significantly broadened ^1^H linewidth compared to the uncrosslinked control. This substantial line broadening demonstrates a dramatic restriction in the segmental mobility of the PE chains and faster transverse relaxation. This molecular‐level rigidification confirms that the PE chains are heavily tethered within a newly formed covalent network, perfectly corroborating the high‐temperature rubbery plateau observed in the DMA. In stark contrast, the *F_1_* linewidths corresponding to all PP signals (the methine carbon at 45 ppm, methylene carbon at 26 ppm, and methyl carbon at 21 ppm) remain virtually identical between the control and the vitrimer. The preservation of these narrow proton linewidths dictates that the PP chains maintain their highly mobile, pre‐extrusion dynamic state. This confirms that the PP domains do not participate in the silyl ether crosslinking network, thus validating our macroscopic hypothesis that the tertiary radicals in PP predominantly undergo competing chain scission rather than bimolecular grafting.

### 3.5 Thermal Properties and Phase Behavior (DSC Analysis)

Differential scanning calorimetry (DSC) was employed to investigate the melting transitions and crystalline morphology of the pure and blended post‐consumer PO networks. The second heating thermograms for the pc HDPE, mixed pc H‐P blends, and pc PP systems are presented in Figure S6, with the corresponding cooling cycles detailed in Figure S7.

In the pure pc HDPE system, the neat control exhibits a sharp melting endotherm at 134.4°C and a crystallization exotherm at 115.9°C. Upon reactive modification (+V+T), both transitions undergo a noticeable depression (***T*_m_** drops to 132.2°C; ***T*_c_** drops to 109.4°C) alongside significant peak broadening and a visible reduction in the melting endotherm's area. Crucially, this reduction in the enthalpy of melting (***∆H*_m_**) distinctly indicates a proportional decrease in the overall degree of crystallinity (***X*_c_**) within the polymer. This decrease in crystallinity occurs because the covalent silyl ether undergoes crosslinking to form permanent pinning points in the melt, thereby significantly restricting the translational mobility of the HDPE chains. This hindered mobility requires a higher degree of thermal undercooling to initiate nucleation and ultimately results in the formation of smaller, less perfect crystalline lamellae. Conversely, the pure pc PP systems experience only minor thermal shifts, as the dominant β‐scission pathways generate structural defects rather than a constraining continuous network.

The thermal profiles of the binary blends (75:25 and 25:75 H‐P) clearly display two distinct sets of melting and crystallization peaks, confirming that the HDPE and PP phases remain thermodynamically immiscible. Crucially, the thermal disruption in these vitrimeric blends is highly asymmetrical. In both the HDPE‐rich and PP‐rich formulations, the polyethylene transitions are drastically suppressed. For instance, in the 25:75 (+V+T) vitrimer, the HDPE melting peak shifts downward by over 10°C, and its corresponding crystallization exotherm nearly collapses into a weak shoulder. Meanwhile, the PP transitions (***T*_m_** ∼163°C, ***T*_c_** ∼122°C) remain relatively stable and sharp. This disparate thermal response provides compelling macroscopic evidence that the radical grafting and dynamic crosslinking are highly selective, localizing predominantly within the HDPE domains while leaving the PP crystalline structures largely undisturbed.

### 3.6 Mechanical Performance

To evaluate the macroscopic impact of the dynamic covalent network on the material's structural integrity, uniaxial tensile testing was performed. The mechanical profiles, specifically the tensile strength, Young's modulus, and elongation at break for the neat controls, the VTMS‐grafted (+V), and the fully formulated (+V+T) vitrimers are summarized in Figure S9. The data reveal a classic transition from ductile thermoplastic behavior to constrained, thermoset‐like mechanics.

**FIGURE 4:**
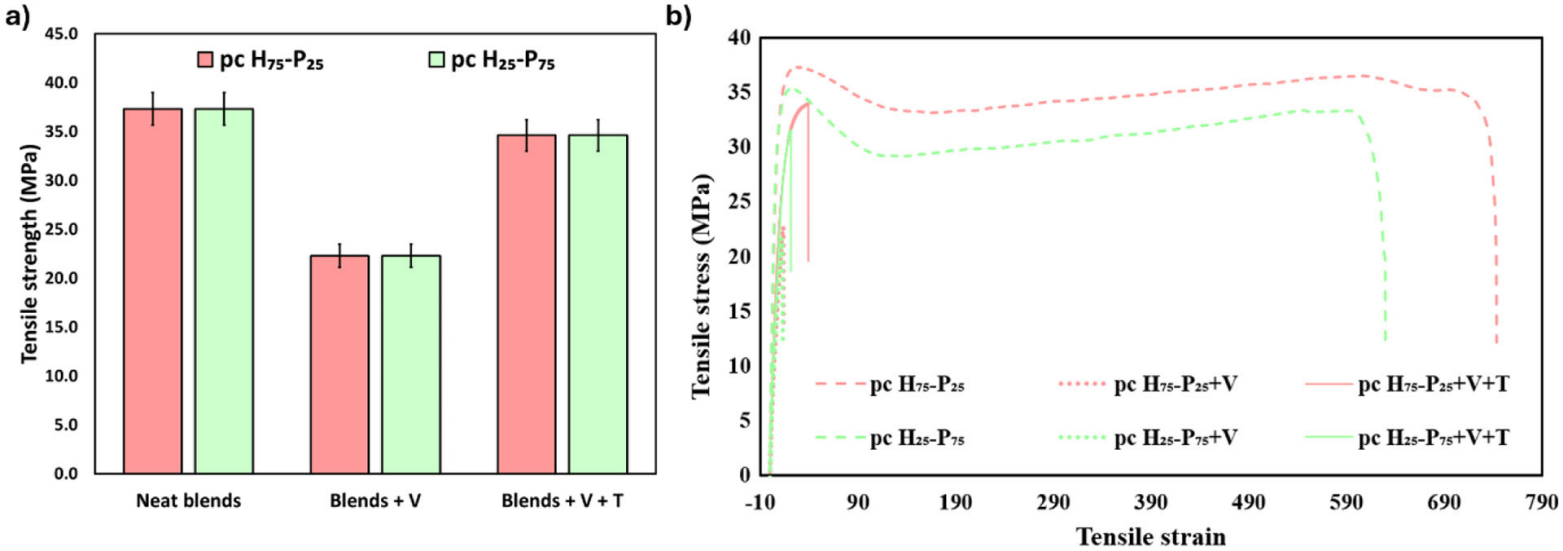
Macroscopic mechanical properties of the upcycled blends. (a) Ultimate tensile strength comparison of the neat blends, grafted vitrimeric blends (Blends + V), and fully formulated vitrimers (Blends + V + T) for the pc H_75_‐P_25_ and pc H_25_‐P_75_ systems. (b) Representative stress‐strain curves for the corresponding HDPE‐rich (pc H_75_‐P_25_) and PP‐rich (pc H_25_‐P_75_) systems.

The neat post‐consumer blends exhibit robust initial tensile strengths ranging from ∼37 MPa. Upon the introduction of the radical initiator and crosslinker (+V), a significant deterioration in tensile strength is observed across almost all systems (e.g., dropping to ∼22 MPa for both the HDPE‐rich and PP‐rich blends). This degradation can be attributed to uncontrolled macroradical side reactions, including chain scission and irreversible dead‐end coupling, which introduce structural defects into the polymer matrix during extrusion. Crucially, the incorporation of the nitroxide radical scavenger (+V+T) facilitates a remarkable recovery in tensile strength for the polyethylene‐containing systems. The pc HDPE‐rich, and PP‐rich vitrimers recover to approximately 35 MPa (Figure 4a and Figure S9a). This confirms that the nitroxide (T) effectively regulates the radical kinetics, trapping intermediates to minimize degradation and promoting a more uniform, stress‐bearing silyl ether network. Interestingly, the pure pc PP (+V+T) system shows a sharp decline in strength (∼28 MPa), further corroborating that in pure PP, radical initiation predominantly drives β‐scission, physically degrading the polymer backbone rather than building a supportive network.

The Young's modulus data strongly aligns with the morphological findings obtained from the DSC analysis. The neat blends are highly stiff (with Young's moduli ranging from ∼560 to ∼750 MPa). Following network formation (+V and +V+T), the modulus drops significantly across all formulations, stabilizing in the ∼300–400 MPa range (Figure S9b). This reduction in stiffness is a direct macroscopic consequence of the ***T*_m_** depression and loss of crystallinity (***X*_c_**) observed in the DSC thermograms. Because the silyl ether crosslinks restrict chain folding and disrupt crystalline perfection, the resulting materials possess a higher amorphous fraction, rendering them slightly more flexible and less stiff than their purely thermoplastic counterparts.

The most profound mechanical evidence of successful network formation is observed in the ductility of the materials. The neat post‐consumer controls are highly ductile, exhibiting massive plastic deformation with elongation at break values ranging from 200% (pc HDPE) to well over 800% (PP‐rich blend). In stark contrast, the fully crosslinked vitrimers (+V and +V+T) experience a catastrophic plunge in elongation, uniformly dropping below 100% (and often below 50% for the blended systems, see stress‐strain curves in Figure 4b and Figure S9c). This severe embrittlement at room temperature is a hallmark characteristic of covalently crosslinked thermosets [51]. The silyl ether linkages act as permanent topological anchor points, severely restricting the polymer chains from sliding past one another under tensile strain. While this sacrifices the extreme ductility of the raw waste plastics, it successfully yields a dimensionally stable, creep‐resistant network that is capable of maintaining its integrity at elevated temperatures (as previously demonstrated by the DMA rubbery plateau).

### 3.7 Melt Reprocessability and Mechanical Performance of MSW‐Simulated Blends

**FIGURE 5:**
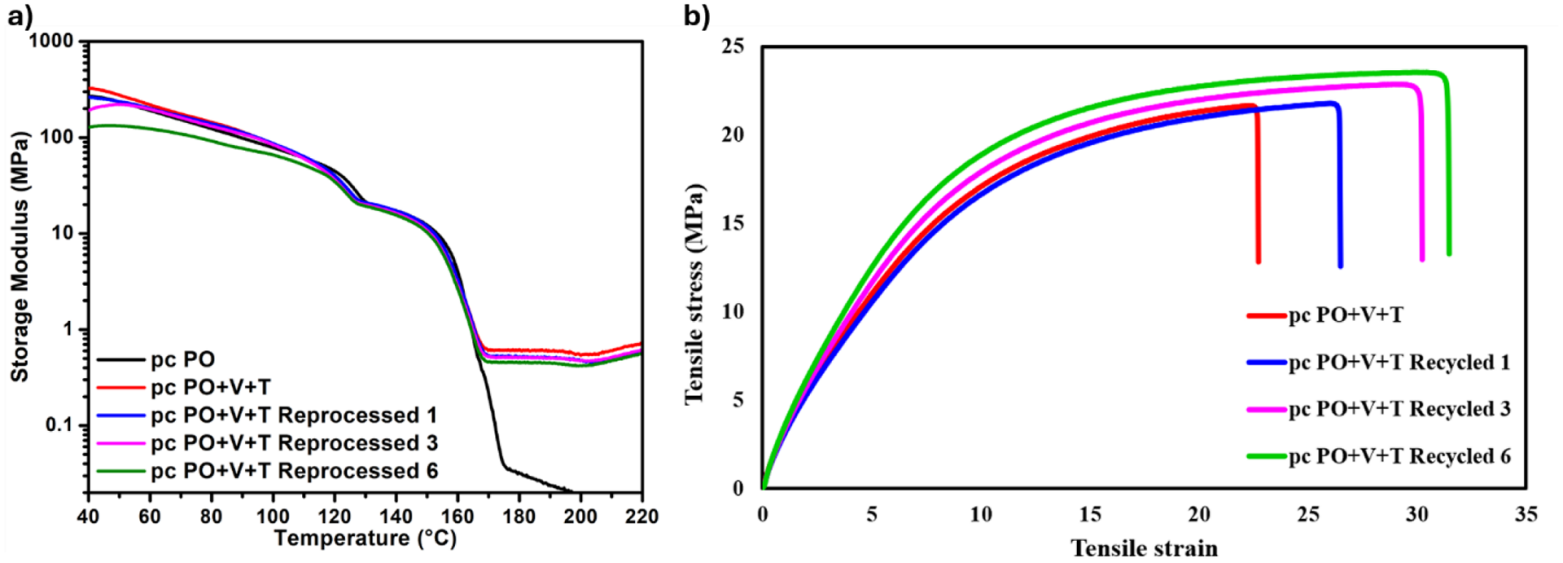
Evaluation of macroscopic reprocessability and network retention for the fully formulated post‐consumer vitrimer (pc PO+V+T). (a) Storage modulus curves demonstrating the retention of the high‐temperature rubbery plateau across six continuous melt‐extrusion cycles, contrasted against the complete melt‐flow failure of the uncrosslinked waste blend (pc PO). (b) Representative stress‐strain curves of the pc PO vitrimer across successive recycling generations.

To evaluate the true industrial viability of this upcycling strategy, a highly complex post‐consumer polyolefin (pc PO) mixture was formulated to simulate actual municipal solid waste, comprising 27.7% HDPE, 36.3% LLDPE/LDPE, and 36.0% PP by weight. The thermomechanical performance and dynamic reprocessability of this fully formulated vitrimer (pc PO+V+T) were evaluated via dynamic mechanical analysis along with tensile testing (Figure 5).

The defining advantage of this material over a thermoset is its capacity for dynamic covalent bond exchange. To demonstrate this, the fully formulated pc PO+V+T vitrimer was subjected to repeated melt‐extrusion cycles. As depicted in Figure 5a, the thermomechanical integrity of the material is remarkably preserved across six continuous reprocessing steps. Following a minor initial drop in the storage modulus after the first cycle, likely attributable to network homogenization and mechanical shearing, the subsequent cycles (1 through 6) exhibit exceptional stability, with the storage modulus stabilizing near 0.4 to 0.5 MPa. This sustained profile unequivocally confirms that the silyl ether crosslinks rapidly undergo associative exchange at the 190°C processing temperature, allowing the mixed MSW plastic to be repeatedly recycled without destroying the underlying thermoset‐like architecture.

To complement the thermomechanical data, tensile testing was conducted on the reprocessed extrudates (Figure 5b). The pc PO vitrimer demonstrates exceptional mechanical resilience, retaining a highly stable ultimate tensile stress between 22 and 24 MPa across all six cycles (Table S2). Notably, a modest but progressive increase was observed across successive cycles not only in the elongation at break (from 21% in the initial formulation to 33% after six cycles), but also in the tensile strength and Young's modulus. We tentatively attribute this concurrent improvement to a slight reduction in the effective crosslink density upon repeated high‐temperature processing, which affords the polymer chains greater mobility to reorganize and crystallize. The resulting increase in crystallinity reinforces the semi‐crystalline matrix, accounting for the gradual rise in tensile strength and modulus.

### 3.8 Thermal Stability and Thermomechanical Benchmarking

**FIGURE 6:**
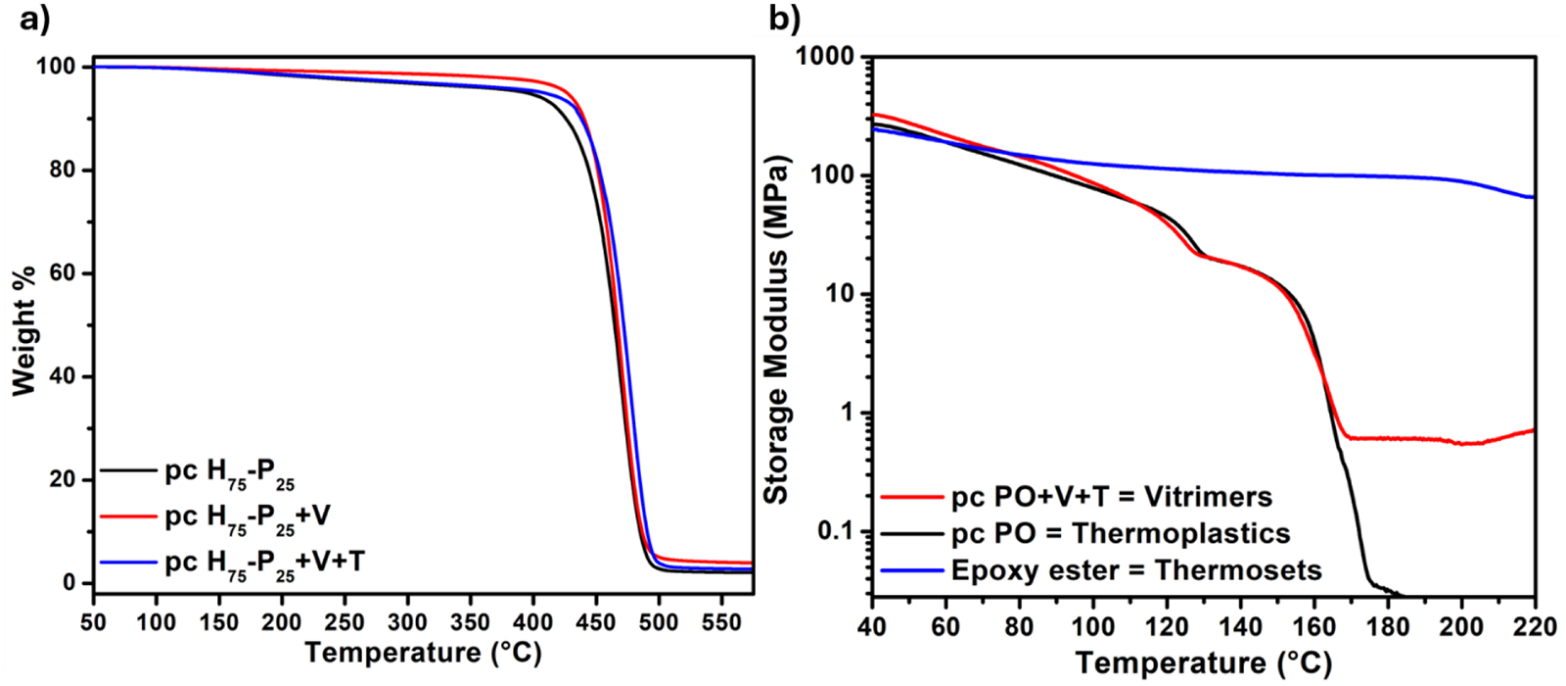
(a) Thermogravimetric analysis of the optimal pc H_75_‐P_25_ system, confirming that the vitrimer architecture maintains high thermal stability. (b) The DMA storage modulus curves compare the post‐consumer polyolefinic vitrimers to their own uncrosslinked precursor (pc PO), representing thermoplastic melt behavior, and the epoxy ester, representing a permanent thermoset.

Thermogravimetric analysis (TGA) of the highest‐performing pc H_75_‐P_25_ formulation (Figure 6a) confirmed that the fully formulated vitrimer maintains exceptional thermal stability, exhibiting degradation onset temperatures well above 400°C, ensuring that the material can safely withstand repeated high‐temperature melt‐reprocessing cycles. This is a crucial finding, as it confirms that the vitrimers remain highly stable at both their service temperatures and their elevated melt‐processing temperature (190°C). All samples demonstrate a characteristic single‐step thermal degradation profile, which corresponds to the random chain scission of the PO backbones. In these systems (Figure 6a and Figure S10a–d), the degradation curves for the crosslinked networks (+V and +V+T) closely overlap with the neat controls. This behavior indicates that the introduction of dynamic silyl ether linkages does not adversely alter the fundamental thermal degradation kinetics of the polymer matrix. A minor residual char yield (∼2%–5%) is observed at 550°C across most samples. This non‐combustible residue is primarily attributed to inorganic fillers and pigments that are inherently present in post‐consumer plastic waste [52], alongside trace silica residue derived from the BTMSPA crosslinker.

To contextualize the thermomechanical performance boundaries of the upcycled MSW vitrimer, its storage modulus profile was benchmarked against the uncrosslinked precursor (pc PO) and a permanent structural thermoset (epoxy ester) (Figure 6b). As expected, the epoxy ester exhibits classic thermoset behavior, maintaining a highly rigid modulus (∼100 MPa) across the entire tested temperature range. On the contrary, the uncrosslinked pc PO thermoplastic completely yields to melt flow and loses structural integrity near 160°C. The upcycled pc PO+V+T vitrimer occupies a distinct intermediate regime: above its melting transition, it successfully establishes a high‐temperature rubbery plateau stabilizing between 0.5 and 0.8 MPa. This profile dictates that the material currently behaves as a lightly crosslinked elastomer, placing its mechanical standing far from the rigid structural regime of a permanent epoxy thermoset, but significantly above the high‐temperature limits of uncrosslinked polyolefins. While this demonstrates a clear high‐temperature advantage, the current rubbery modulus indicates that the degree of crosslinking can be further improved.

## 4 Conclusion

This study, for the first time, validated the successful formation of vitrimers from post‐consumer PE and PP blends via nitroxide‐mediated silyl ether chemistry. Optimization of the mixed waste streams revealed that the HDPE‐rich 75:25 (HDPE/PP) blend vitrimer offers the highest performance, yielding the best thermomechanical properties. 2D WISE solid‐state NMR confirmed that dynamic crosslinking is highly phase‐selective, which directly explains why maximizing the PE concentration yields the strongest network, as it effectively tethers the PE domains while suppressing the degradation of the dynamically inactive polypropylene phases. Thermomechanical benchmarking confirmed that the upcycled material behaves as a lightly cross‐linked elastomer, retaining dimensional stability at temperatures above 180°C, where uncrosslinked polyolefins would otherwise succumb to melt flow. This specific thermomechanical profile, combined with the retention of dynamic bond exchange across six complete melt‐extrusion cycles, significantly reduces the environmental burden of PO accumulation by offering a truly circular, high‐performance material lifespan. This work suggests that future research in sustainable polymer chemistry should focus on leveraging this phase‐selective dynamic chemistry in even broader ranges of municipal solid waste, alongside investigating advanced reactive compatibilization techniques to actively reinforce the non‐participating polypropylene domains within these complex heterogeneous blends.

## Author Contributions

**Subhaprad Ash**: conceptualization, investigation, Writing – original draft, methodology, validation, visualization, formal analysis, data curation. **Rishi Sharma**: data curation. **Li Xie**: data curation. **Muhammad Rabnawaz**: funding acquisition, writing – review and editing, supervision, resources, conceptualization, project administration.

## Conflicts of Interest

The authors declare no conflicts of interest.

## Supporting information




**Supporting File**: marc70373‐sup‐0001‐SuppMat.docx.

## Data Availability

The data that support the findings of this study are available from the corresponding author upon reasonable request.
