# Supplementary material for: Reprocessable Vitrimers From Post‐Consumer Polyolefin Blends: Elucidating Phase‐Selective Dynamic Networks
Source: Macromol Rapid Commun. 2026 Jul 20;47(17):e70373. doi: 10.1002/marc.70373 (PMC13542787; doi:10.1002/marc.70373)
Supplement: Supplementary file 1 [file MARC-47-e70373-s001.docx]

**Supporting Information**

for

**Reprocessable Vitrimers from Post-Consumer Polyolefin Blends: Elucidating Phase-Selective Dynamic Networks**

*Subhaprad Ash^a, b^, Rishi Sharma^a^, Li Xie^b^ and Muhammad Rabnawaz^a,c^**

^a^ School of Packaging, Michigan State University, East Lansing, MI 48824-1223, USA

^b^ Department of Chemistry, Michigan State University, East Lansing, MI 48824-1223, USA

***Corresponding Author:** [Muhammad Rabnawaz](about:blank)

**Tel.:** +1-517-432-4870

**E-mail:** [rabnawaz@msu.edu](about:blank);

**Address:** School of Packaging, Michigan State University, 448 Wilson Road, East Lansing, Michigan 48824-1223, USA.

**1. FT-IR and Silane Content Analysis**

**Figure S1.** FT-IR transmittance spectra confirming silyl ether network formation across varying post-consumer polyolefin (PO) compositions: **(a)** pc HDPE, **(b)** pc (H_75_-P_25_), **(c)** pc (H_25_-P_75_), and **(d)** pc PP series. The spectra validate the successful incorporation of silyl ether linkages (1092 cm^-1^) via reactive extrusion.

**Figure S2.** FT-IR transmittance spectra of mixed post-consumer polyolefin compositions having HDPE, LDPE/LLDPE, and PP.

**Figure S3.** Relative silane content analysis before and after boiling xylene extraction to assess grafting efficiency. Values represent the FT-IR absorbance ratio of the silane group (A_1092_) against the stable polyolefin CH_2_ internal reference (A_1461_).

**Table S1.** FT-IR absorbances and silane content analysis for HDPE-rich and PP-rich post-consumer vitrimeric samples.

| **Samples** | **A_1092_ (A.U.)** | **A_1461_ (A.U.)** | **A_1092_ / A_1461_ for samples extracted in xylene** | **A_1092_ / A_1461_ for unextracted samples** | **Reduction in silane relative to unextracted samples (%)** |
| --- | --- | --- | --- | --- | --- |
| **pc H₇₅-P₂₅+V** | 0.0530 | 0.1686 | 0.3143 | 0.3666 | 14.27 |
| **pc H₇₅-P₂₅+V+T** | 0.0424 | 0.1056 | 0.4019 | 0.4116 | 2.34 |
| **pc H₂₅-P₇₅+V** | 0.0579 | 0.2594 | 0.2233 | 0.3426 | 34.84 |
| **pc H₂₅-P₇₅+V+T** | 0.0757 | 0.2457 | 0.3082 | 0.3985 | 22.65 |

**2. Dynamic Mechanical Analysis (DMA)**

**Figure S4.** Thermomechanical analysis of the synthesized dynamic networks via dynamic mechanical analysis (DMA). Storage modulus (***E'***) as a function of temperature is plotted for **(a)** pc HDPE, and **(b)** pc PP systems.

**3. Solid State NMR**

**Figure S5.** 1D CP-MAS spectrum of the control polyolefin (PO) blend and PO vitrimer.

**4. DSC Analysis**

**Figure S6.** Differential scanning calorimetry (DSC) heating thermograms of the pure and blended post-consumer polyolefin systems: **(a)** pure pc HDPE, **(b)** 75:25 pc HDPE/PP, **(c)** 25:75 pc HDPE/PP, and **(d)** pure pc PP. The curves illustrate the melting behavior and endothermic transitions (***T*_m_**) of the neat control blends (black) compared to the silyl ether-grafted (+V, red) and fully formulated vitrimer networks (+V+T, blue).

**Figure S7.** DSC cooling thermograms detailing the crystallization behavior of the post-consumer polyolefin systems: **(a)** pure pc HDPE, **(b)** 75:25 pc HDPE/PP, **(c)** 25:75 pc HDPE/PP, and **(d)** pure pc PP. The curves demonstrate the restricted chain mobility (lowered ***T*_c_**) induced by the silyl ether network, most notably in the PE-containing domains of the vitrimer formulations (+V and +V+T).

**Figure S8.** Differential scanning calorimetry (DSC) heating **(a)** and cooling **(b)** thermograms of the mixed PO MSW simulated systems

**5. Tensile properties**

**Figure S9.** Mechanical properties of the neat post-consumer polyolefins compared to their modified vitrimer networks (+V and +V+T). The bar charts depict: **(a)** tensile strength, **(b)** Young’s modulus, and **(c)** elongation at break %. The data highlights the recovery of tensile strength via nitroxide stabilization (+V+T) and the dramatic reduction in elongation, signifying the transition from a highly ductile thermoplastic to a restricted, covalently crosslinked network.

**Table S2.** Mechanical properties of the pc PO vitrimer across successive recycling generations.

| **Sample code** | **Tensile strength (MPa)** | **Young's Modulus (MPa)** | **Elongation at break (%)** |
| --- | --- | --- | --- |
| **pc PO** | 38.4 ± 0.9 | 483 ± 13 | 1144.2 ± 108.4 |
| **pc PO+V+T** | 20.5 ± 1.5 | 282 ± 5 | 20.6 ± 3.7 |
| **pc PO+V+T Recycled 1** | 21.6 ± 0.3 | 277 ± 12 | 25.4 ± 1.4 |
| **pc PO+V+T Recycled 2** | 22.2 ± 1.1 | 294 ± 5 | 24.4 ± 4 |
| **pc PO+V+T Recycled 3** | 22.5 ± 0.5 | 303 ± 7 | 28.9 ± 1.6 |
| **pc PO+V+T Recycled 4** | 23.6 ± 0.5 | 309 ± 6 | 32.8 ± 0.3 |
| **pc PO+V+T Recycled 5** | 23.7 ± 0.2 | 313 ± 4 | 37.3 ± 4 |
| **pc PO+V+T Recycled 6** | 23.5 ± 0.5 | 313 ± 12 | 33.3 ± 3.8 |

*Note: The terms “Reprocessed 1”, “Reprocessed 2”, “Reprocessed 3”, “Reprocessed 4”, “Reprocessed 5”, and “Reprocessed 6” refer to samples that had been reprocessed 1, 2, 3, 4, 5, and 6 times, respectively.*

**6. TGA Analysis**

**Figure S10.** Thermogravimetric analysis curves evaluating the thermal stability of the neat post-consumer (pc) resins, grafted intermediates (+V), and fully formulated vitrimers (+V+T). Formulations evaluated include: **(a)** pure pc HDPE, **(b)** pure pc PP, **(c)** the PP-rich binary blend (pc H_25_-P_75_), and **(d)** the MSW-simulated complex mixture (pc PO).
